# Glycans modify mesenchymal stem cell differentiation to impact on the function of resulting osteoblasts

**DOI:** 10.1242/jcs.209452

**Published:** 2018-02-15

**Authors:** Katherine M. Wilson, Alistair M. Jagger, Matthew Walker, Estere Seinkmane, James M. Fox, Roland Kröger, Paul Genever, Daniel Ungar

**Affiliations:** 1 Department of Biology, University of York, York YO10 5DD, UK; 2 Department of Physics, University of York, York YO10 5DD, UK

**Keywords:** Glycan processing, Hydroxyapatite, Kifunensine, Osteogenesis, PI3K signalling

## Abstract

Glycans are inherently heterogeneous, yet glycosylation is essential in eukaryotes, and glycans show characteristic cell type-dependent distributions. By using an immortalized human mesenchymal stromal cell (MSC) line model, we show that both *N*- and O-glycan processing in the Golgi functionally modulates early steps of osteogenic differentiation. We found that inhibiting O-glycan processing in the Golgi prior to the start of osteogenesis inhibited the mineralization capacity of the formed osteoblasts 3 weeks later. In contrast, inhibition of *N*-glycan processing in MSCs altered differentiation to enhance the mineralization capacity of the osteoblasts. The effect of *N*-glycans on MSC differentiation was mediated by the phosphoinositide-3-kinase (PI3K)/Akt pathway owing to reduced Akt phosphorylation. Interestingly, by inhibiting PI3K during the first 2 days of osteogenesis, we were able to phenocopy the effect of inhibiting *N*-glycan processing. Thus, glycan processing provides another layer of regulation that can modulate the functional outcome of differentiation. Glycan processing can thereby offer a novel set of targets for many therapeutically attractive processes.

## Abstract (precis)

**Summary:** Both *N*- and O-glycan processing modulate MSC differentiation early during osteogenesis to influence mineral formation. Inhibition of *N*-glycan processing increases mineralization.

## INTRODUCTION

Glycosylation is a ubiquitous post-translational modification of proteins. Glycans synthetized in the eukaryotic secretory pathway are credited with several examples of modifying specific protein functions, such as the folding, stability and targeting of secretory proteins (Ihrke et al., 2001; Kingsley et al., 1986). Moreover, the importance of glycan processing for the development of complex life is well recognized, given many described developmental defects caused by aberrant glycosylation (Hennet and Cabalzar, 2015). It is also well documented that protein-linked glycan compositions undergo large changes during differentiation events in mammals, thereby giving rise to large cell type-dependent variations in glycan profiles (An et al., 2012; Hamouda et al., 2013; Hasehira et al., 2012; Wilson et al., 2016). However, it is largely unknown whether these glycosylation changes are functionally contributing to the differentiation process itself, potentially altering the function of the differentiated cells, or are mere bystanders of cell-specification processes.

Differences in glycosylation are established during glycan processing, mainly in the Golgi. The inherent heterogeneity of glycosylation, ensured by the 200-plus enzymes that add or remove monosaccharides, thereby makes it difficult to assess the contributions of specific glycans or even glycan types to cellular function. Glycans can be lipid- or protein-linked, the latter classified as O-linked if the glycan is attached to Ser/Thr or *N*-linked for Asn-attached glycans. For *N*-glycans, a mannose-rich chain – called an oligomannose glycan – is established in the endoplasmic reticulum (ER) and trafficked with its carrier protein to the Golgi. Here, mannoses are trimmed in the *cis*/*medial*-Golgi before addition of *N*-acetylglucosamine (GlcNAc) residues in the *medial-*Golgi. The added GlcNAc residues initiate branches that form the basis for hybrid and complex *N*-glycans. Complex *N*-glycan chains contain galactose, fucose and lactosamine modifications, and are commonly terminated with sialic acids. If initial mannose trimming by the enzyme mannosidase I is inhibited, only oligomannose *N*-glycans will be present (Elbein et al., 1990). In contrast, inhibition of the mannosidase II enzyme will favour hybrid glycan formation (Gross et al., 1983). In mammals, O-glycans are largely divided into mucin-types and the more specialized glycosaminoglycans, although other minor types are also present. Details of mucin type O-glycan processing are less well understood than those for *N*-glycans. O-glycan biosynthesis is initiated by an *N*-acetylgalactosamine (GalNAc) residue, which, in the case of mucin-type O-glycans, is elaborated with galactose and GlcNAc in branched arrangements to form one of four core structures (Ungar, 2009). These core structures are further modified by the addition of lactosamine, sialic acid and fucose modifications, similar to, but often distinct from, *N*-glycans. The processing of mucin type O-glycans can be inhibited by GalNAc analogues that competitively inhibit the addition of monosaccharides downstream of the initial GalNAc (Kuan et al., 1989). Besides the expression and activities of glycan-processing enzyme subsets, their localization within the Golgi also markedly influences glycan profiles. Enzyme localization is determined through vesicular sorting, mediated by the conserved oligomeric Golgi (COG) tethering complex (Miller and Ungar, 2012). COG mutations have been shown to result in glycosylation defects in various organisms (Bailey Blackburn et al., 2016; Belloni et al., 2012; Kingsley et al., 1986; Struwe and Reinhold, 2012; Whyte and Munro, 2001; Wu et al., 2004) by affecting multiple different glycosylation pathways (Kingsley et al., 1986; Spessott et al., 2010). This is due to the mis-sorting of Golgi proteins (Oka et al., 2004), which affects both their final locations and steady state levels (Fisher and Ungar, 2016).

There is limited evidence for a functional contribution of glycans to the differentiation process, and this is restricted to terminal glycan modifications. For example, glycan features such as lactosamine and fucosylation have been implicated in the self-renewal of stem cells, and, by implication, in the prevention of differentiation (Hamouda et al., 2013; Kumar et al., 2013; Sasaki et al., 2011). A particular focus has been on the role of terminal sialic acids. Sialidase treatment has been shown to reduce osteogenesis (Tateno et al., 2016) and regulatory T-cell differentiation (Lin et al., 2015). In contrast, sialylation has been found to inhibit human embryonic stem cell differentiation (Alisson-Silva et al., 2014). In addition, immune development also involves sialylation that influences major histocompatibility complex binding (Moody et al., 2001).

The differentiation of mesenchymal stem cells (MSCs) is a good model to investigate the contributions of glycans to the differentiation process. The glycan profiles of these stem cells and their progeny have been well documented. For example, in MSCs, ∼45% of the *N*-glycans are oligomannose with 55% complex *N*-glycans, this is altered to 30% oligomannose and 70% complex *N*-glycans in osteoblasts (Wilson et al., 2016). Osteoblasts differentiate from MSCs and are responsible for bone generation. MSCs can receive pro-osteogenic signals, as well as anti-adipogenic and anti-proliferative cues, to promote differentiation into osteoblast precursors. Runt-related transcription factor 2 (Runx2) is a key transcription factor that regulates osteogenesis (Ducy et al., 1997). It induces the expression of several osteoblast specific genes, for example, those encoding α1 collagen and bone sialoprotein (BSP; also known as IBSP). A large body of work has implicated various signalling pathways in osteogenic differentiation and subsequent mineralization (Cook and Genever, 2013). In particular, TGF-β signalling [for example, through bone morphogenetic protein 2 (BMP2), Lee et al., 2000], Wnt signalling (via Wnt3a; Quarto et al., 2010) and several FGF pathways (Su et al., 2014) all feed into osteogenesis. The involvement of individual pathways is often controversial. For example, the phosphoinositide-3-kinase (PI3K) signalling pathway has been implicated both in promoting (Hamidouche et al., 2009) and antagonizing (Kratchmarova et al., 2005) osteogenesis, dependent on the activation state of the pathway within specific stages of osteogenic differentiation.

Here, we find that alteration of either *N*- or O-glycan processing in the Golgi fundamentally influences osteogenic differentiation of an immortalized human clonal MSC line. While inhibiting O-glycan processing inhibited functional osteogenic differentiation, interestingly, the inhibition of an early step in *N*-glycan processing altered differentiation to ultimately promote more mineralization in the formed osteoblasts. This change in differentiation is due to altered PI3K-mediated signalling in the first days of osteogenic differentiation, implicating protein-linked glycans in the fine-tuning of signalling to influence cellular differentiation.

## RESULTS

### Genetic disruption of the glycosylation synthesis pathway alters the *N*- and O-glycan profile of hTERT-MSCs

**Fig. 1.**
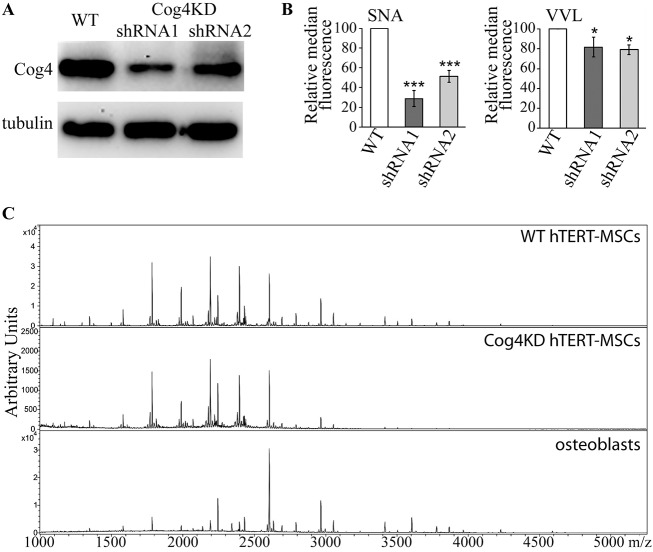
**Cog4 knockdown in MSCs causes glycosylation defects.** (A) Immunoblot of WT and Cog4KD Y101 hTERT-MSC lysates probed for Cog4. Anti-tubulin was used as a loading control. (B) WT and Cog4KD Y101 hTERT-MSCs were stained with 10 µg/ml FITC-tagged VVL or 10 µg/ml biotin-labelled SNA followed by 5 µg/ml FITC–streptavidin, and the fluorescence intensity of 10^5^ cells was measured by FACS. Shown are the median fluorescence intensities of the KD cell lines relative to WT. Results are mean±s.e.m. (*n*=3). (C) Mass spectrometric *N*-glycan profiles of FANGS-released *N*-glycans from WT (top) and Cog4KD (shRNA1, middle) Y101 hTERT-MSCs, and osteoblasts derived from Y101 hTERT-MSCs (bottom). Detailed annotated spectra of the WT and osteoblast lines have been published in Wilson et al., 2016, and are reproduced here to serve as a comparison for the Cog4KD cells. *P<0.05, ***P<0.001.

Altering glycosylation enzyme localization in the Golgi by interfering with vesicular sorting has the potential to broadly perturb several different glycan biosynthetic pathways. This can be used to globally assess the function of glycans in a cell biological process. Such a perturbation was achieved by knocking down a subunit of the COG vesicle tethering complex, Cog4, by means of RNA interference. MSCs exist in a heterogeneous population of bone marrow stromal cells, including partially differentiated osteoprogenitors, from which they are hard to distinguish. To help circumvent any issues associated with MSC heterogeneity, here we used a highly characterized human clonal MSC line. Y101 cells were previously immortalized by transduction with human telomerase reverse transcriptase (hTERT), and represent a reproducible model of *in vitro* differentiation (James et al., 2015). Importantly, glycosylation of the Y101 line is unlikely to be altered due to immortalization, as comparison of three independent hTERT-MSC clones with different phenotypes (Y101, Y201, Y202) has shown no significant differences in their *N*-glycan profiles (Wilson et al., 2016). hTERT-MSC Y101 clones expressing one of two different Cog4-specific shRNAs were generated (Cog4KDshRNA1 and Cog4KDshRNA2). Both Cog4KD MSC lines have over 60% reduction in Cog4 protein levels compared to that in wild-type (WT) controls (Fig. 1A). Glycosylation alterations were first investigated by measuring lectin staining via flow cytometry. As expected, both the sialic acid-binding *Sambucus nigra* lectin (SNA) and the GalNAc-binding *Vicia villosa* lectin (VVL) showed reduced staining in Cog4KD lines (Fig. 1B; Fig. S1). These indicate changes in mucin type O-glycan and likely also *N*-glycan biosynthesis. A recent report with a more detailed description of the changes in the O-glycan profile of Cog4KD MSCs confirms these differences, indicating a global alteration of mucin type protein O-glycosylation (Skeene et al., 2017). To identify more specific changes in *N*-glycan processing, Cog4KD hTERT-MSCs were harvested, and total cellular *N*-glycans isolated by filter-aided *N*-glycan separation (FANGS; Abdul Rahman et al., 2014). Following permethylation, the glycans were analysed by matrix-assisted laser desorption ionization–time of flight (MALDI-TOF) mass spectrometry. The *N*-glycan profile of Cog4KD cells is highly similar to that of WT hTERT-MSCs [Fig. 1C, compared here to the published (Wilson et al., 2016) WT and osteoblast spectra]. No significant differences are seen when individual glycans of >0.02% abundance are quantified (Table S1), or when the relative abundance of the different glycan classes is calculated (Fig. S2). The abundance of higher-mass complex glycans above about 2700 *m*/*z* is visibly lower in the Cog4KD spectrum. However, this difference is not statistically significant when the relative abundances from multiple repeats are summed up. Interestingly though, the same class of higher-mass glycan species dominates the profile of osteoblasts differentiated from the hTERT-MSCs (Wilson et al., 2016 and Fig. 1C). Given the successful alteration of protein glycosylation in MSCs, *in vitro* osteogenesis was used as a model to assess the impact of glycan biosynthesis modulation on cell differentiation.

### Glycosylation impacts on the mineralization behaviour of differentiating osteoblasts

**Fig. 2.**
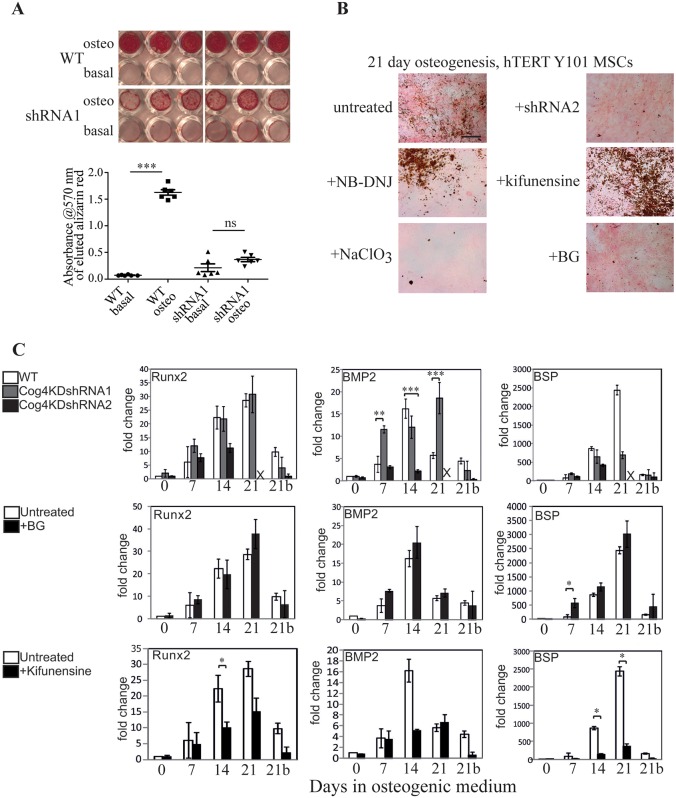
**Altered glycan processing affects the function of MSC derived osteoblasts.** (A) Alizarin Red staining of WT or Cog4KD Y101 hTERT-MSC cells following a 21 day differentiation experiment in osteogenic medium. Quantification of the eluted Alizarin Red stain is shown in the graph below the image. Results are mean±s.d. (*n*=6). (B) ALP and von Kossa staining of Y101 cells or Y101 cells stably expressing a Cog4-specific shRNA (shRNA2) following incubation in osteogenic medium with the indicated inhibitors for 21 days. NB-DNJ, *N*-butyldeoxynojirimycin; BG, benzyl-O-GalNAc. Scale bar: 100 µm. (C) Real-time qPCR analysis of mRNA expression levels of indicated genes in the Y101 WT or shRNA KD cells. Medium was supplemented with the indicated inhibitors 2 days prior to the start of and until the end of the differentiation experiments. Averages of triplicate measurements were normalized to the day zero control. Data represent the mean of the mean of three technical repeats each for two independent biological replicates (each normalized independently to its own control) with s.e.m. shown. The bar positions marked with ‘X’ are from the 21 day shRNA2 samples that peeled off. All qPCR experiments in Figs 2C and 3B were performed in parallel, and used the same untreated WT as control. This control is replicated in all three rows of 2C as well as 3B for clarity. *P<0.05, **P<0.01, ***P<0.001 (compared to the control samples are marked with asterisks); all other changes are non-significant.

As previously reported, Y101 hTERT-MSCs cultured in osteogenic medium for 3 weeks undergo differentiation into osteoblasts, and produce hydroxyapatite mineral deposits (James et al., 2015). At the same time there is a dramatic change in the *N*-glycan profile of the cells (Wilson et al., 2016), which could serve as MSC-specific markers. To assess whether the glycans are also functionally important during differentiation, the glycosylation defective Cog4KD Y101 cells were subjected to differentiation conditions. After culturing in osteogenic medium for 3 weeks, WT cells showed productive osteogenic differentiation, as visualized by Alizarin Red staining (Fig. 2A), pink alkaline phosphatase (ALP) staining and numerous von Kossa-stained brown dots, representing mineralized calcium phosphate deposits (Fig. 2B). In contrast, the Cog4KD lines showed minimal Alizarin Red staining and very few von Kossa-stained deposits, attesting to largely failed mineralization, although ALP staining was still present (Fig. 2A; Fig. 2B, top right). Quantification of the eluted Alizarin Red stain confirmed the strong mineralization defect (Fig. 2A, graph).

**Fig. 3.**
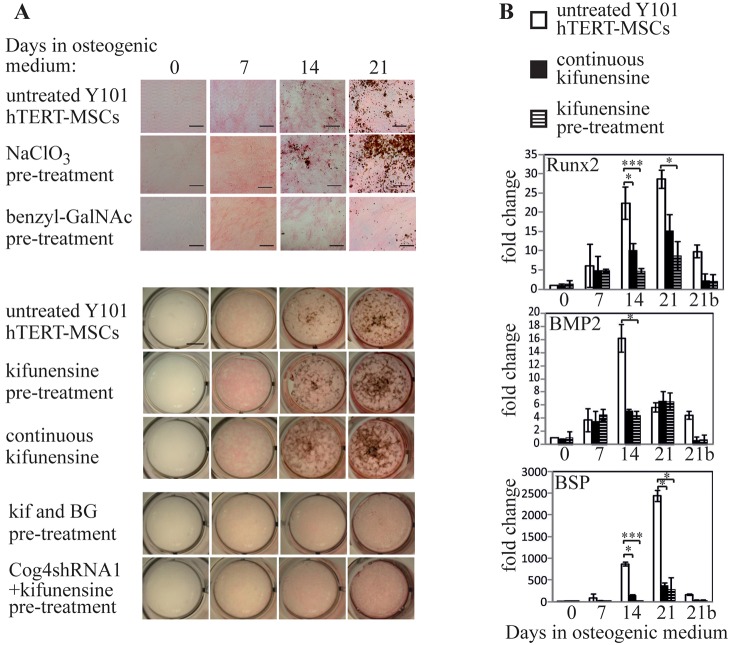
***N*- and O-glycan processing mediate early steps in differentiation.** (A) WT Y101 hTERT-MSCs were pre-treated with the indicated inhibitors for 48 h before differentiation was induced in osteogenic medium in the absence of inhibitors for 21 days (inhibitors were included for the full 21 days for comparison where indicated as ‘continuous’). ALP and von Kossa staining was performed on the indicated days. The images in the last row show Cog4KD cells rather than WT. Scale bar: 100 µm (top three rows), 5 mm (bottom five rows). (B) Real-time qPCR experiments performed as in Fig. 2C with Y101 cells cultured in osteogenic medium in the continuous presence of kifunensine following a 48 h pre-treatment or pre-treated with kifunensine for 48 h followed by incubation in medium without kifunensine. The +kif data are repeated from Fig. 2 for clarity. Data represent averages of mean±s.e.m. of three technical repeats each for two independent biological replicates. *P<0.05, ***P<0.001 (compared to the control samples), all other changes are non-significant.

Cog4 depletion alters a number of different glycosylation pathways (Fisher and Ungar, 2016), including *N*-glycan, O-glycan (Kingsley et al., 1986) and glycolipid (Spessott et al., 2010) biosynthesis. We next sought to address which of these specifically affect differentiation of the Y101 hTERT-MSCs. Cells were therefore cultured in the presence of various glycan biosynthesis inhibitors and their differentiation assessed. We have previously published that inhibiting complex *N*-glycan biosynthesis by using swainsonine did not affect mineralization (Wilson et al., 2016). Inhibiting glycolipid biosynthesis by using *N*-butyldeoxynojirimycin (NB-DNJ; Fig. 2B, middle left) had no effect on ALP and von Kossa staining either. To investigate further the contributions of the *N*-glycan processing pathway, the mannosidase I inhibitor kifunensine was used, which completely inhibits *N*-glycan processing in the Golgi, but has no effect on the proliferation rate of cells (Fig. S2A–C,E). Interestingly, addition of kifunensine during osteogenesis resulted in visibly more abundant von Kossa staining, indicating increased mineralization (Fig. 2B, middle right). These observations imply that complex and hybrid *N*-glycans and glycolipid glycans are not responsible for the observed defects in Cog4-depleted cells. In contrast, inhibiting O-glycan biosynthesis phenocopied the effects of Cog4 depletion by inhibiting mineralization. Differentiating cells in the presence of the proteoglycan sulfation inhibitor NaClO_3_ (Fig. 2B, bottom left) or the mucin type O-glycan synthesis inhibitor benzyl-O-GalNAc (BG; Fig. 2B, bottom right), led to positive pink ALP staining, without significant amounts of brown von Kossa staining. Inhibiting the processing of mucin type O-glycans again had no effect on cell proliferation (Fig. S2B,D,E), although the GAG sulfation inhibitor NaClO_3_ at the used concentration did slow down the proliferation of cells upon prolonged incubation (Fig. S2F).

While mineralization is a clear functional consequence of osteogenic differentiation, its absence does not necessarily mean a complete ablation of the osteogenic differentiation programme. As another test, real-time quantitative PCR (qPCR) assays were performed investigating the expression levels of three mRNAs as markers of osteogenic differentiation (Kulterer et al., 2007): the transcription factor Runx2, the growth factor BMP2, and the crystal nucleator BSP. Despite observing a clear loss of mineralization in both Cog4KD lines, there was little difference in the expression patterns of Runx2 between WT and the shRNA1 line. While the fold-changes for shRNA2 are different, the trends are consistent with what was seen in both shRNA1 and WT cells (Fig. 2C). Levels of BMP2 and BSP mRNA did not show sharp changes at 21 days in shRNA-treated cells as seen in WT, and the effects were opposite for the two markers (compared to the shRNA, higher in WT for BSP, lower in WT for BMP2; Fig. 2C, top row). Even less different compared to controls were BG-treated cells, as no differences in the levels of the tested markers could be seen (Fig. 2C, middle row). For the drug treatments, we investigated two further genes, the transcription factor osterix (also known as SP7) as an additional early marker, and the crystallization factor osteocalcin as a late marker (Kulterer et al., 2007). While neither of these showed a statistically significant difference compared to the untreated controls, the trend in the osteocalcin gene expression was consistent with the lack of mineralization (Fig. S3). Indeed, comparing samples on separate individual days, the decrease in osteocalcin expression on day 7 and 28 was statistically significant. This change in osteocalcin expression could be responsible for the reduced mineralization seen with BG, something future studies could address. Surprisingly, following kifunensine treatment, which enhanced mineralization (Fig. 2B), the mRNA levels of Runx2, BSP and osteocalcin were consistently lower throughout osteogenesis than in control cells. BMP2 expression did increase in-line with the control after 7 days, but failed to reach the maximal induction at day 14 (Fig. 2C, bottom row). Collectively these qPCR-based findings may imply that the main impacts of Cog4 depletion, as well as kifunensine- and BG-treatments, are on the process of mineralization rather than differentiation per se, but they do not rule out a direct involvement in controlling aspects of cell differentiation.

### *N*- and O-glycosylation directly impact on MSC differentiation

The results presented so far implicate both *N*-glycan and mucin-type O-glycan processing in osteoblast functionality, but do not provide evidence for their involvement in differentiation itself. We noticed that most inhibitors had to be added for a period of 48 h prior to differentiation for effects to be observed, while shorter pre-treatments had no effect. We reasoned that specific glycan types could be generated or depleted in this pre-differentiation period, which could prime the cells for altered differentiation and following this, osteoblast activity. To test whether specific glycan types are involved in early differentiation decisions, cells were treated with glycosylation inhibitors for 48 h prior to addition of osteogenic medium. Osteogenic differentiation was then continued in the absence of glycosylation inhibitors for 21 days.

Inhibiting proteoglycan sulfation is known to be important for osteogenesis (Kumarasuriyar et al., 2009 and Fig. 2B). However, pre-treatment of Y101 MSCs with the sulfation inhibitor NaClO_3_ was not sufficient to influence mineralization in the same way as continuous treatment. Pre-treated cells showed ALP and von Kossa staining that was indistinguishable from controls (Fig. 3A, second row). In contrast, BG and kifunensine pre-treatments (Fig. 3A, third and fifth rows) had the same effect on decreasing and increasing mineralization respectively, as continuous treatment with the inhibitors. Given the markedly reduced expression of osteogenic marker mRNA levels during continuous kifunensine treatment, we wondered whether this effect was also recapitulated during the pre-treatment-only regime. Indeed, the pattern of expression levels of Runx2, BMP2 and BSP mRNAs were very similar in kifunensine pre-treated and continuously treated hTERT-MSCs (Fig. 3B), and similar results were obtained for osteocalcin and osterix as well (Fig. S3). Together, the mineralization and qPCR data indicate altered MSC osteogenic differentiation when protein glycosylation is modified.

To determine the epistatic relationship between the *N*- and O-glycan-processing pathways during MSC differentiation, hTERT-MSCs were co-treated with kifunensine and BG for 48 h in basal medium, prior to subjecting them to osteogenic medium in the absence of the glycan-processing drugs for 21 days (Fig. 3A, seventh row). The lack of positive von Kossa staining at the end of the 21 day differentiation following dual treatment indicates that O-glycan processing is dominant over *N*-glycan processing. Interestingly, treatment of Cog4KD hTERT-MSCs with kifunensine also did not rescue the mineralization defect (Fig. 3A, bottom row) suggesting that disruption of the O-glycan synthesis pathway could likely contribute to the altered MSC phenotype caused by Cog4 knockdown.

### Enhancement of mineralization does not alter the ultrastructure of the formed mineral

**Fig. 4.**
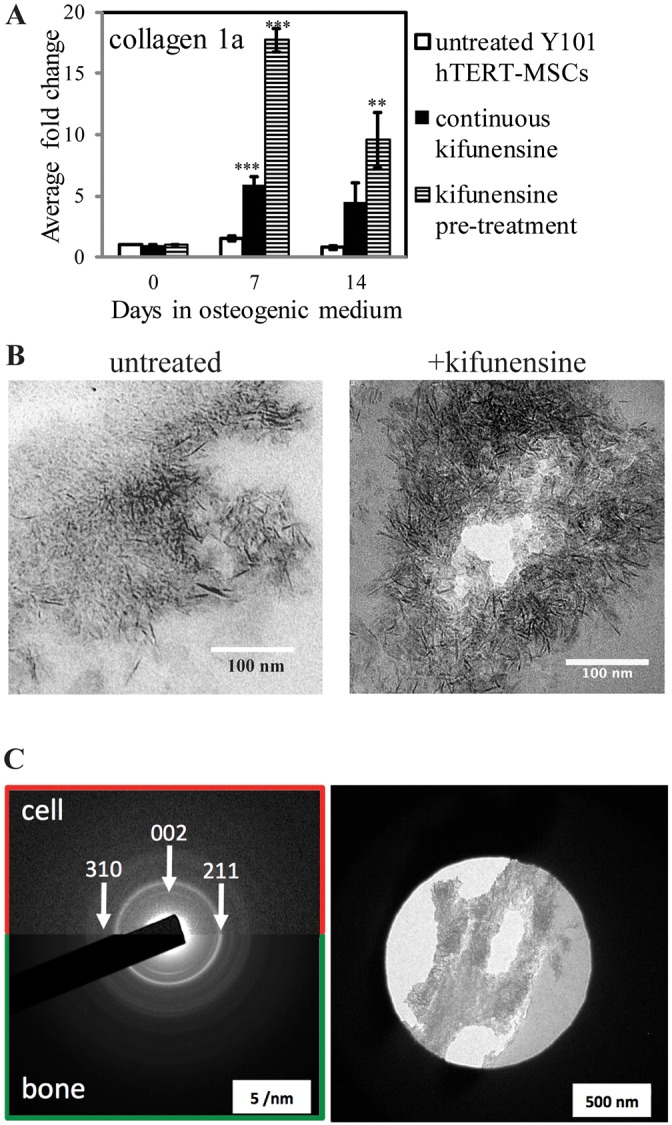
**Mineralization enhancement by kifunensine yields well-structured mineral deposits.** (A) Real-time qPCR analysis of collagen type 1 gene expression in Y101 cells incubated in osteogenic medium with the indicated inhibitor treatments. Experiments performed as for Fig. 2C, but for 14 days only. (B) Transmission electron micrographs of 90 nm thin sections prepared from Epon Araldite-embedded Y101 cell samples following 21 days of osteogenic differentiation. Kifunensine treatment was for 48 h prior to start of differentiation only. (C) Electron diffraction images of a bone sample (bottom half) and a sample of the resin-embedded Y101 cells differentiated following kifunensine pre-treatment (top half). The positions of three diffraction rings characteristic for hydroxyapatite are indicated with arrows. The diffraction image of the cells was taken from the region shown in the right side panel. **P<0.01, ***P<0.001 (compared to the control samples are marked with asterisks); all other changes are non-significant.

The enhanced mineralization potential of kifunensine-treated MSCs could be particularly interesting from a therapeutic angle. However, the reduced levels of established osteogenic markers observed by qPCR during the same differentiation experiments could indicate a non-conventional differentiation process, which could potentially cause abnormal or artefactual mineral formation. ALP is important for mineralization as it generates the phosphate required for hydroxyapatite. Histological staining did show ALP to be present in kifunensine-treated cells (pink staining in ALP- and von Kossa-stained images in Figs 2B and 3A), and this was confirmed by western blot analysis (Fig. S4A). On the blot, the level of ALP, as well as its apparent molecular mass are lower upon continuous kifunensine treatment, but both of these are reverted to normal in samples that have only been pre-treated with the drug. Importantly though, total enzymatic activity is not significantly different in continuously kifunensine-treated compared to control samples (Fig. S4B). A possible explanation for this is that altered glycosylation may impact on ALP enzymatic activity. In conclusion, it is not simply enhanced phosphate generation that is responsible for the increased mineralization in kifunensine-treated cells. Key for the formation of mineral with similar properties to that naturally formed in bone is the control of hydroxyapatite organization through collagen fibrils. Importantly, further real-time qPCR analysis of kifunensine pre-treated and continuously treated hTERT-MSCs revealed that collagen type I is significantly upregulated by both treatments (Fig. 4A), suggesting a potential physiological route for increased mineralization.

Collagen type I is a major constituent of bone, and mutations to type I collagen are associated with the brittle bone disease osteogenesis imperfecta (Sykes et al., 1986). Importantly, apatite nanocrystals nucleate within the collagen gap regions to form an aligned arrangement parallel to the collagen fibrils (Wang et al., 2012). This was investigated using bright-field transmission electron microscopy to characterize the size and shape of the apatite crystals, and selected area electron diffraction (SAED) to unambiguously identify the apatite mineral. High-magnification transmission electron micrographs of unstained thin sections following resin-embedding of both kifunensine pre-treated and control MSCs following three weeks of osteogenesis were collected. The appearance of the mineral-covered areas was very similar between the kifunensine-treated and untreated samples apart from a generally higher density of the mineral in the drug-treated ones (Fig. 4B). The form and size of the needle-shaped hydroxyapatite crystals was measured as 5.40±0.80 nm (mean±s.d.; *n*=15) for control and 5.14±1.16 nm (*n*=17) following kifunensine treatment. This is in good agreement with the ∼5 nm width reported for hydroxyapatite crystals in bone specimens (Rubin et al., 2003). To ascertain that the observed crystals following kifunensine treatment are bona fide apatite crystals, and have the same crystal form as the ones found in bone, SAED experiments were carried out. These showed a strong 002 diffraction ring with a pattern typical of apatite (Fig. 4C, compare with Chatzipanagis et al., 2016). Discontinuity of the 002 reflection in the diffraction pattern can determine co-alignment of the apatite nanocrystals, a feature typically found in bone (Chatzipanagis et al., 2016; Rubin et al., 2003). This is generally a difficult experiment in a 2D tissue culture model, as the amount of deposited mineral is usually not sufficient to observe directional bias of the crystals. However, the high density of the mineral deposits generated by kifunensine-treated cells allowed us to observe an incomplete 002 ring. As expected, this effect is more clearly visible in the included diffraction pattern of a bone sample due to the higher amount of ordered mineral in bone compared to the *in vitro* cell model (Fig. 4C, compare top and bottom half). This indicates that in kifunensine-treated cells crystal orientation starts to align with that of the collagen fibrils generally regarded as the sites of nucleation and crystal growth in bone (Weiner and Traub, 1992). These observations suggest that kifunensine treatment increases collagen type 1 expression, thereby facilitating the generation of mineral deposits that possess the correct structure and orientation bias as required for bone formation.

### Identifying the signalling pathway linking *N*-glycan processing and osteogenesis

The results so far suggest that kifunensine acts early in the MSC osteogenic differentiation process. Given that the extracellular portions of signalling pathways are usually mediated by glycoproteins, we wondered whether the effects of kifunensine treatment could be attributed to a particular signalling pathway. MSC proliferation and osteogenic differentiation have been associated with several major signalling pathways, including Wnt/β-catenin, TGF-β–BMP/Smad and tyrosine kinase receptor-mediated (e.g. FGFR) pathways. We compared the activation status of these pathways in the first few days of MSC osteogenic differentiation in the presence of a 48 h pre-treatment with kifunensine, BG and both inhibitors, or in the absence of a drug treatment. We reasoned that the pathway responsible for the mineralization enhancing effects of kifunensine should have an altered response upon kifunensine pre-treatment, when compared to untreated or BG pre-treated samples. As expected for Wnt signalling following the induction of osteogenesis, active β-catenin levels did increase, but there was no marked difference between the various treatments (Fig. S5A). Similarly, phosphorylation of the MAP kinases ERK1 and ERK2 (ERK1/2, also known as MAPK3 and MAPK1, respectively) was not markedly different upon kifunensine treatment when compared to untreated or BG-treated osteogenic samples (Fig. S5B). Although treatment with both inhibitors did lead to reduced ERK1/2 activation after 7 days (Fig. S5B, last lane), this was not further investigated given the lack of difference between the other three treatment groups. Moreover, investigating the TGF-β pathway, we could not detect significant levels of phosphorylated Smad2 or Smad3 under any of the treatment conditions.

**Fig. 5.**
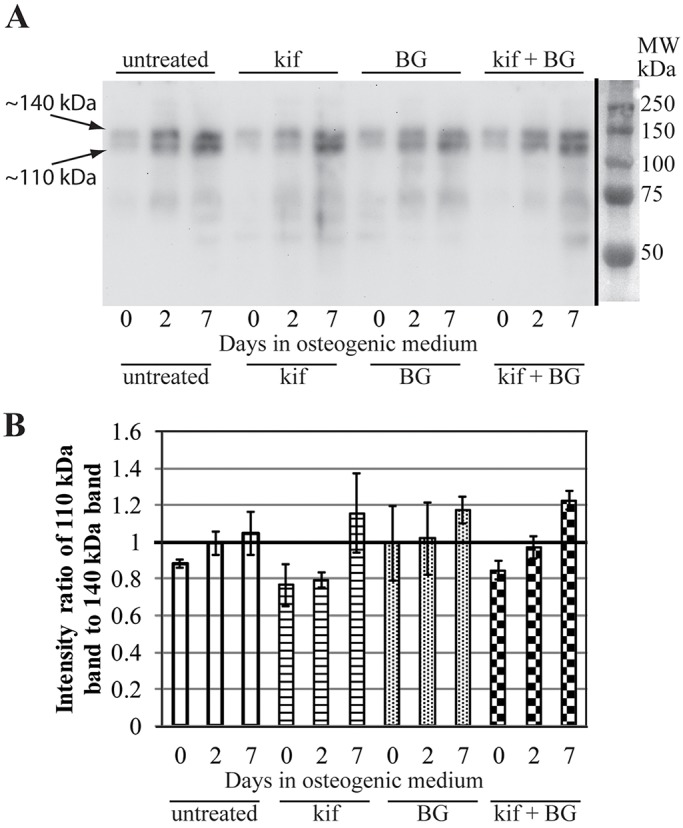
**Altered phosphotyrosine signalling early in osteogenesis upon kifunensine treatment.** (A) Y101 cells cultured in osteogenic medium for 0, 2, 7 days were treated with the indicated inhibitors for 48 h prior to the start of differentiation. Total cell lysates were separated on 10% SDS-PAGE and immunoblots probed with the phosophotyrosine-specific PY20 antibody. (B) The intensities of the two major bands between the 100 and 150 kDa molecular mass markers (marked with arrows, with the apparent molecular masses indicated) were quantified by using ImageJ, and their ratio charted. Results are mean±s.e.m. for two independent experiments.

To test for receptor tyrosine kinase signalling, the abundances of phosphotyrosine (pTyr)-containing proteins were compared between the treatment groups during the first week of osteogenesis (Fig. 5A). While there are differences in the overall intensity of pTyr staining over time, which are independent of the applied drug treatment, the ratio of the two most intense bands seen between the 100 and 150 kDa molecular mass markers is specifically altered. At day two of differentiation in the kifunensine-treated sample the lower band of the doublet decreases in intensity relative to the upper band (Fig. 5A,B). Although this change was not statistically significant, we decided to follow up on this clear trend. We speculated that the lower of these two bands could represent the p110 subunit of PI3K. This speculation is somewhat substantiated by findings of Kratchmarova and colleagues, based on the molecular mass and related observations about platelet derived growth factor (PDGF)-induced tyrosine-phosphorylated proteins in MSCs (Kratchmarova et al., 2005). Interestingly, that study implicated the PI3K pathway in the PDGF-dependent inhibition of osteogenesis (Kratchmarova et al., 2005).

**Fig. 6.**
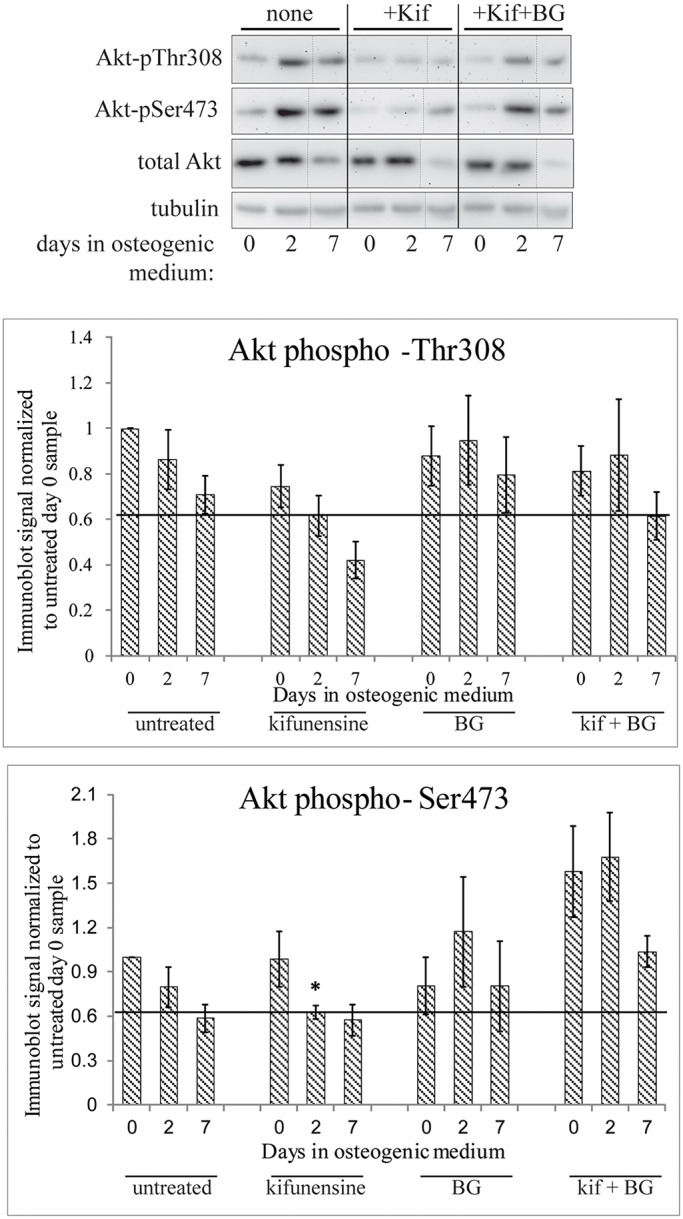
**Altered Akt activation during early osteogenic differentiation following kifunensine treatment.** Cell lysates prepared as in Fig. 5A were immunoblotted for total Akt as well as for Thr308 and Ser473 phosphorylation on Akt. Band intensities were quantified by means of ImageJ and averaged following normalization of the phosphorylation-specific signals to the tubulin and total Akt signals, followed by normalization to the day zero untreated sample. Dotted lines indicate where the blot was cut and pasted to move two lanes from the same blot next to each other. All bands shown in one row are taken from the same image. Horizontal lines in the bar charts were drawn to highlight the levels of phospho-Akt in kifunensine pre-treated day 2 samples. Error bars show s.e.m. for *n*=4 (all pThr308 samples), *n*=6 (untreated, kifunensine and BG treated pSer473 samples), *n*=3 (double treated pSer473 samples). Asterisk marks the statistically significant (*P*<0.05) reduction of pSer473 staining in the kifunensine treated day 2 sample.

Lowered PI3K phosphorylation would lead to a change in the levels of phosphorylated Akt family proteins (hereafter Akt), a downstream effector of PI3K (Franke et al., 1995). Total Akt levels as well as the phosphorylation state of the two main sites (Thr308 and Ser 473) both change over the course of the first week of differentiation even in the absence of any other treatments (Fig. 6, compare with no treatment). However, when the phosphorylation states were carefully quantified and normalized to both total Akt and the housekeeping protein tubulin, a change upon kifunensine treatment did emerge. Both the Thr308 and the Ser473 residues of Akt had a lower level of phosphorylation 2 days after the start of differentiation in kifunensine-treated hTERT-MSCs compared to the untreated and BG-treated cells (Fig. 6). In the case of Akt Thr308 phosphorylation, this difference even persisted at the 7 day time point. None of the differences were statistically significant after only four experimental repeats. Given the stronger effect on pSer473 phosphorylation at 2 days, we increased the sample size for this phosphorylation site to six, which indeed showed a statistically significant reduction in phosphorylation after 2 days specifically in the kifunensine pre-treated sample. This supports the hypothesis that inhibition of PI3K signalling could be the mechanism by which kifunensine treatment alters osteogenesis and enhances consequent mineralization.

**Fig. 7.**
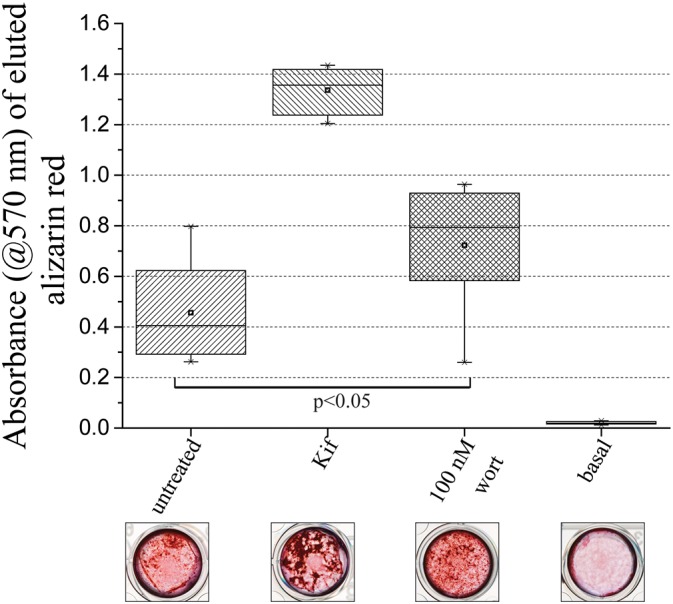
**PI3K inhibition can phenocopy the effect of kifunensine treatment.** Y101 cells were grown in osteogenic medium or control basal medium for 21 days and stained with Alizarin Red. Cells were either untreated, pre-treated with kifunensine for 48 h, or treated with 100 nM wortmannin for the first 2 days of the differentiation period. Images of representative stained wells are shown below the quantification of the eluted Alizarin Red. The box represents the 25–75th percentiles, and the median is indicated by the line. The mean is indicated by a square. The whiskers show the 5–95th percentiles. *n*=8 (*n*=6 for untreated).

Based on the above observations the PI3K inhibitor wortmannin was used to attempt to phenocopy the effects of kifunensine. We first verified that 100 nM wortmannin indeed resulted in a significant reduction of Akt phosphorylation in the presence of osteogenic medium (Fig. S6). We then reasoned that the 48 h pre-treatment with the glycosidase inhibitor would have altered the glycosylation state of signalling components by the time differentiation was initiated. This then would have had a transient effect on the signalling machinery during the first few days of differentiation. Therefore, wortmannin was applied during the first 2 days of differentiation onto MSCs that were not treated with kifunensine, and following 3 weeks of differentiation the cells were stained with Alizarin Red to allow quantification of the formed mineral deposits. Treating Y101 cells with 100 nM wortmannin during the first 2 days of differentiation significantly increased Alizarin Red staining, and by inference mineralization (Fig. 7), similar to what was seen with kifunensine treatment.

## DISCUSSION

Our study provides evidence for the functional involvement of Golgi-based glycan processing in the early steps of cellular differentiation. Restricting mannosidase I inhibition to the 2 days prior to the start of differentiation increased mineral formation. It is evident that the effect of kifunensine pre-treatment is transient, for example the altered glycosylation of ALP seen upon continuous treatment disappears when only pre-treatment is applied (Fig. S3A). These data argue that it is indeed the differentiation programme itself that glycosylation influenced early on rather than merely affecting the molecular function of the factors responsible for mineral formation, which themselves appear later during differentiation (Cook and Genever, 2013). Given that swainsonine treatment did not alter the mineralization behaviour of osteoblasts (Wilson et al., 2016) but kifunensine treatment significantly increased it, it is not simply the lack of complex glycans driving the enhancement. Similarly, it is not just the lack of terminal sialylation, an alteration that has previously been implicated in differentiation events (Alisson-Silva et al., 2014; Moody et al., 2001), which drives this differentiation mechanism. Future studies will have to address exactly which *N*-glycan species are needed for enhanced differentiation, although this could well involve a range of glycans. The *N*-glycan profiles of three different hTERT-MSC lines (Y101, Y201 and Y202) are virtually indistinguishable (Wilson et al., 2016), while their differentiation characteristics are clearly different (James et al., 2015). Therefore, *N*-glycans almost certainly play a modulatory role for differentiation rather than a deterministic one. Interestingly, a second type of glycan processing, that of mucin type O-glycans, also modulates differentiation, in this case inhibiting the generation of functional osteoblasts that are capable of mineral deposition. This is also due to an early event, as in the case of mannosidase I inhibition.

Which molecular factors could be responsible for the effects of altered glycan processing on differentiation of this model cell system? *N*-glycan processing has been shown to influence the signalling behaviour of receptors such as EGFR (Sato et al., 2001) and cytokine receptors (Partridge et al., 2004). In these cases, the effect of the glycan is on receptor endocytosis, and mostly depends on complex *N*-glycan branching (Lau et al., 2007). This mechanism by itself is less likely to be relevant given the differences observed between inhibiting mannosidase I and II, which both eliminate complex glycan branching. In contrast, ICAM-1-mediated signalling was significantly altered by limiting its glycans to an oligomannose form, which directly influenced the interactions with intracellular partners (Scott et al., 2013). Such a mechanism could potentially be more relevant in our system. The effects of glycosylation on receptor/growth factor function are generally conveyed by lectins that specifically bind to the presented glycans. Recently the lectin Clec11a (osteolectin) was shown to promote osteogenesis of MSCs (Yue et al., 2016). The consensus carbohydrate-binding motif of Clec11a does not conform to either a galactose specific Gln-Pro-Asp nor a mannose specific Glu-Pro-Asn sequence (Drickamer, 1992). This makes it difficult to implicate Clec11a in a mechanism of kifunensine-dependent increased mineralization before its monosaccharide specificity has been investigated.

While pinpointing the specific glycoprotein(s) responsible for mediating the consequences of altered glycan processing is beyond the scope of this study, it was possible to delineate affected signalling pathways and their outputs. Using the clonal Y101 MSC line, rather than a variable mixed MSC population, has the advantage of well-defined differentiation characteristics (James et al., 2015) in order to study fundamental cell biological questions. It is interesting though that most of the tested qPCR markers do not follow the trend expected from the functional output of mineralization. For example, O-glycan modulation, which suppresses mineralization, has no effect on Runx2, BMP2, BSP and osterix. In contrast, kifunensine in part suppresses the upregulation of these markers although it promotes increased mineralization. The late marker osteocalcin could potentially explain the effects of O-glycan inhibition, as it does show a trend for lower expression, but it again cannot explain the effects of kifunensine. The much reduced upregulation of the markers in the case of kifunensine could imply a shift of biosynthetic capacity that would instead, for example, be used to generate collagen, which we did find to be upregulated. We propose the rather speculative possibility that in Y101 cells collagen levels are rate-limiting for mineralization whereas other differentiation induced factors are not. In line with this reasoning, ALP activity is not limiting here for increased mineralization. The rebalancing of resources (from Runx2, BMP2 and BSP towards collagen) through altered glycan processing could tie in with the established role of the glycan processing machinery in translating the nutrient state of the cell into growth cues (Lau et al., 2007).

As Y101 cells are a subclone of MSCs, it is difficult to assess the overall importance of the uncovered effects on the PI3K pathway for osteogenesis in a primary MSC mixture. Both pro- and anti-osteogenic effects of PI3K signalling have previously been reported. The studies on this have investigated PI3K signalling from different angles. For example, PI3K was described as an essential pro-osteogenic signalling component downstream of BMP2 (Ghosh-Choudhury et al., 2002), Wnt3a (Ling et al., 2010) and integrin (Hamidouche et al., 2009) signalling. In contrast, PI3K inhibition was shown to release the inhibition of osteogenesis conveyed by PDGF activation (Kratchmarova et al., 2005). Thus, the PI3K pathway could act either positively or negatively on osteogenesis, dependent on the circumstances. It is also apparent that the MAPK pathway, which often shares cell surface receptors with the PI3K pathway can be regulated in opposite ways or even independently of PI3K (Yokota et al., 2014). What is common in all previous studies though, is that they investigated PI3K signalling throughout the studied differentiation program. We can now add a further piece to this puzzle by showing that a short transient inhibition of PI3K at the start of osteogenesis can alter differentiation to ultimately enhance mineralization in the Y101 MSC line. This modulatory effect of PI3K signalling is in fact a likely conduit for glycans to influence differentiation. Thus fine-tuning the balance in signalling pathways is something that altered glycoforms can potentially accomplish. While our understanding of these processes is limited at the moment to our model cell line, representing a subpopulation of MSCs, they could still be important in the context of varying glycosylation between individuals, in diseases or ageing. Interestingly, many individuals suffering rare congenital glycosylation disorders have symptoms consistent with bone malformations (Coman et al., 2008).

## MATERIALS AND METHODS

Chemicals were from Sigma and cell culture reagents from Invitrogen unless otherwise indicated.

### Cell culture, osteogenesis and inhibitor treatments

Y101 hTERT-MSCs (James et al., 2015) were cultured in basal medium [Dulbecco's modified Eagle's medium (DMEM), high glucose, pyruvate, no glutamine, supplemented with 10% fetal bovine serum (FBS), 1% penicillin/streptomycin and 1% Gluta-Max-I]. To induce osteogenic differentiation basal medium was supplemented with 50 μg/ml ascorbic acid, 5 mM β-glycerophosphate and 10 nM dexamethasone, and changed every 3–4 days for the indicated period.

Cog4KD hTERT-MSCs were generated by using MISSION shRNA lentiviral particles (Sigma, TRCN0000180098 designated shRNA1 and TRCN0000149947 designated shRNA2) according to the manufacturer's instructions. A multiplicity of infection (MOI) of 1 was used for transduction. Single-cell-derived clones were selected using 2 μg/ml puromycin. The Cog4 MISSION constructs TRCN0000146949, TRCN0000423404 and TRCN0000443798 were also used but did not result in significant Cog4 knockdown.

For treatments with glycosylation inhibitors [kifunensine (Kif, Santa Cruz Biotechnology, 2 μg/ml), benzyl-2-acetamido-2-deoxy-D-galactose (BG, 2 mM), NaClO_3_ (25 mM), *N*-butyldeoxynojirimycin (NB-DNJ, Santa Cruz Biotechnology, 150 μM)] cells were cultured in basal medium with addition of the inhibitor for 48 h before the start of differentiation. Following this treatment the inhibitor was added to osteogenic medium where indicated.

### Flow cytometric analysis of lectin binding

All centrifugations were at 450 ***g*** for 5 min. Cells were washed twice with phosphate-buffered saline (PBS), incubated at 37°C for 10 min with washing buffer [0.2% bovine serum albumin (BSA), 5 mM EDTA in PBS], and detached cells centrifuged. The cells were resuspended in PBS, counted and re-pelleted. All following incubations were at 4°C or on ice. Cells were resuspended in PBS at 10^6^ cells/ml, 100 μl of the suspension was incubated for 15 min before adding 100 μl lectin in washing buffer and incubating in the dark for 30 min. Following addition of 1 ml washing buffer and centrifugation, the pellet was resuspended in 100 μl of washing buffer containing 1 μg/ml DAPI for FITC-conjugated lectin (Vector Labs) or 5 μg/ml streptavidin-fluorescein (Vector Labs) for biotinylated lectin and incubated in the dark for 5 min (DAPI, for FITC-lectin) or 30 min (streptavidin–FITC, for biotin-lectin). Streptavidin–FITC was washed out three times before addition of DAPI. Following DAPI incubations, 1 ml of washing buffer was added, and cells were pelleted and resuspended in 400 μl PBS. FACS was performed on a CyAn ADP Analyzer (Beckman Coulter) using the 405 and 488 nm lasers. Cells were gated for forward- and side-scatter to exclude debris and against 405 nm to select live cells. The count versus log 488 nm fluorescence of live cells was displayed as a histogram and the median fluorescence of this histogram used in comparisons between samples.

### Isolation and analysis of *N*-glycans by mass spectrometry

*N*-glycan samples were prepared using FANGS as described previously (Abdul Rahman et al., 2014). Glycans were then permethylated and analysed by mass spectrometry as described previously (Wilson et al., 2016), using an ultraflex III MALDI-TOF mass spectrometer (Bruker). Spectra were analysed using Flex Analysis 3.3 (Bruker) as described previously (Wilson et al., 2016). In brief, after assignment of a glycan structure to the mono-isotopic peak, the intensities of all corresponding isotopic peaks were summed providing the total peak intensity for a given glycan. Total peak intensities normalized to the sum of all peak intensities within a spectrum were averaged between spectra.

### Histological staining

All incubations were at room temperature. Stained wells were imaged with a stereo microscope (Zeiss) or a Leica DMLA upright microscope. Cells for staining were cultured in 24-well plates.

For alkaline phosphatase (ALP) and von Kossa staining, cells were washed twice with PBS before a 5 min incubation in ALP stain solution (0.2 mg/ml naphtol AS-MX in 1% *N*,*N*-dimethylformamide, 1 mg/ml Fast Red TR diluted in 0.1 M Tris-HCl pH 9.2). Following two PBS washes and fixation (4% paraformaldehyde, 5 min), cells were washed with PBS, then with water before incubation in 1% silver nitrate on a light box for 30 min. Following this, cells were washed three times with distilled water, incubated for 5 min with 2.5% sodium thiosulphate, and following two distilled water washes, stored in PBS with 20% glycerol.

For Alizarin Red staining, following two PBS washes and 15 min fixation in 4% paraformaldehyde, cells were washed three times with PBS before a 20 min incubation in 40 mM Alizarin Red S (pH 4.2, reagent filtered and pH adjusted every week). Cells were washed at least five times in tap water until excess stain was removed, then air dried. Stain was eluted following imaging by a 2 h incubation in 10% cetylpyridinium chloride and quantified by measuring absorbance at 570 nm. If the absorbance was too high, all samples were equally diluted in water and re-measured.

### MTT assay

For each cell line tested, 1200 cells were seeded per well, into six wells of a 96-well culture plate. After the indicated number of days, culture medium was replaced with 100 μl fresh medium and 25 μl 3-(4,5-dimethylthiazol-2-yl)-2,5-diphenyltetrazolium bromide (MTT) solution (5 mg/ml in PBS). Plates were then incubated for 3 h at 37°C in 5% CO_2_. MTT solution and medium was removed from wells, and 100 μl of 0.04 M HCl in isopropanol was added to each well. Plates were left to shake at room temperature for 10 min to allow for complete solubilization. Absorbance was then read at 570 nm, and average absorbance (with standard deviation) was used in comparisons.

### Analysis of gene expression by real-time qPCR

RNA was extracted from cells cultured in a well of a six-well plate by using TRIzol (Invitrogen), and suspended in 12 μl nuclease-free water (Hypure, ThermoScientific). RNA was treated with RQ1 RNase-free DNase I (Promega) before using 1 μg for SuperScript II (Invitrogen) catalysed cDNA synthesis. Real-time qPCR was performed with Fast SYBR Green (Applied Biosciences) on a StepOnePlus system (Applied Biosciences) and analysed using StepOne v2.3 software. Gene expression levels were quantified by using the comparative CT (ΔΔCt) method, by normalizing both to the housekeeping gene RPS27a and WT (or untreated) day 0 levels. Primer pairs (given 5′–3′) were: Runx2, AGTGATTTAGGGCGCATTCCT and GGAGGGCCGTGGGTTCT; BMP2, ACTCGAAATTCCCCGTGACC and CCACTTCCACCACGAATCCA; BSP, GAGGAGGAAGAGGAAGGAAATG and TGGTACTGGTGCCGTTTATG; osterix, GCCAGAAGCTGTGAAACCTC and GCTGCAAGCTCTCCATAACC; osteocalcin, AGGGCAGCGAGGTAGTGAAG and AGGGGCAAGGGGAAGAGGAAAG; collagen 1A1, CAAGAACCCCAAGGACAAGAG and CTTGCAGTGGTAGGTGATGGTC; RPS27a, TGGATGAGAATGGCAAAATTAGTC and CACCCCAGCACCACATTCA.

### Analysis of protein expression by western blotting

Sample buffer (150 µl; 5% glycerol, 50 mM Tris-HCl pH 6.8, 50 mM DTT, 1% SDS, 0.7 mM Bromophenol Blue) was used to lyse cells from a well of a 24-well plate. Proteins were separated on 10% gels prior to semi-dry transfer onto polyvinylidene fluoride (PVDF) membrane (ThermoFisher) for 60 min at 20 V using 48 mM Tris-HCl, 39 mM glycine, 20% methanol and 0.0375% SDS as the transfer buffer. Membranes were blocked using PBS with 0.05% Tween-20 and 5% milk (PBSTM) for 1 h (3% BSA was used instead of milk for blots with the anti-pTyr PY20 antibody), then incubated with primary antibody: affinity purified anti-Cog4 (1:500, Miller et al., 2013), anti-tubulin (1:2000, gift from M. Gerard Waters, Princeton University, Princeton, NJ), anti-ALP (1:6000, Santa Cruz Biotechnology sc-137213), anti-active β-catenin (1:2000, Millipore 050665), anti-phosphotyrosine (1:1000, with 3% BSA instead of milk, BD Bioscience clone PY20), anti-Akt (1:1000, Santa Cruz Biotechnology sc-1618), anti Akt-pThr308 (1:1000, Cell Signaling 13038), anti-Akt-pSer473 (1:1000, New England Biolabs 4060P) anti-pSmad2/3 (1:500, Santa Cruz Biotechnology sc-11769), anti-pERK (1:4000, R&D Systems AF1018) in PBSTM overnight at 4°C. Following six 10 min washes with PBSTM, appropriate HRP-linked secondary antibody (1:3000, Bio-Rad) in PBSTM was added for 1 h, then the blot was washed, and imaged on a GeneGenius Chemi-imager (Syngene) after application of Immobilon HRP substrate (Millipore). Quantification was carried out using ImageJ software.

### Electron microscopy

Following 3 weeks of osteogenesis, cells were embedded in Epon Araldite (EMS), and 90 nm thick sections were placed onto 200 mesh carbon-coated copper grids. Grids were imaged on a JEOL 2010 TEM operated at 200 kV with a LaB6 crystal as electron source and an UHR lens for an ultimate spatial resolution of 1.9 Å. Selected area electron diffraction patterns to identify the mineral phase were recorded at a camera length of 0.25 m. Crystal width was quantified by using ImageJ. The distance between points of half-maximum intensity on lines plotted perpendicular to the long axis of the crystals was determined, averaged and then multiplied by two to give crystal width.

### Statistical analysis

Data were analysed using SigmaPlot 12.3 software. First, a normality test (Shapiro–Wilk) and a test of equal variance was performed. For comparison of two groups, a Student's *t-*test was used; for more groups, a one-way ANOVA test was performed, followed by Holm–Sidak post-hoc tests if required. If the data failed the normality or variance tests, a Mann–Whitney test was carried out when two groups were compared, and a Kruskal–Wallis one-way analysis of variance on ranks, followed by Dunn's post-hoc test was carried out if more than one group was being compared. *P<0.05, **P<0.01 and ***P<0.001, ns, non significant.

## Supplementary Material

Supplementary information
