# Supplementary material for: Glycans modify mesenchymal stem cell differentiation to impact on the function of resulting osteoblasts
Source: J Cell Sci. 2018 Feb 15;131(4):jcs209452. doi: 10.1242/jcs.209452 (PMC5868951; doi:10.1242/jcs.209452)
Supplement: Supplementary information [file joces-131-209452-s1.pdf]

Wilson et al.
Supplementary Figure 1.

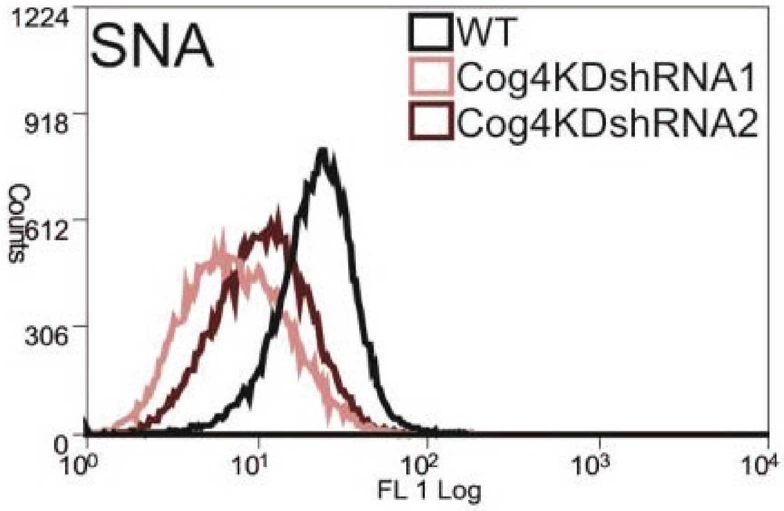

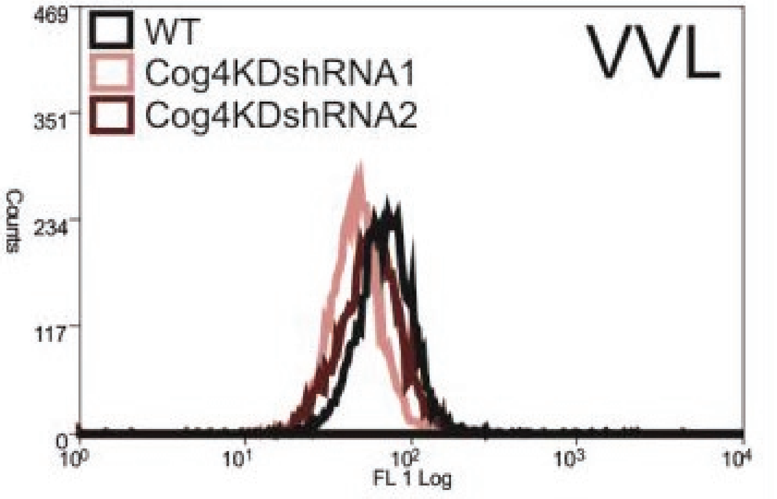

Supplementary Figure 1. Glycan analysis of Cog4KD cells using fluorescent lectins.
WT (black), Cog4KDshRNA1 (pink) and Cog4KDshRNA2 (brown) Y101 hTERT-MSCs were probed with SNA (top) and VVL (bottom) lectins. Histograms show cell counts versus log FITC fluorescence.

Journal of Cell Science • Supplementary information

Wilson et al.
Supplementary Figure 2

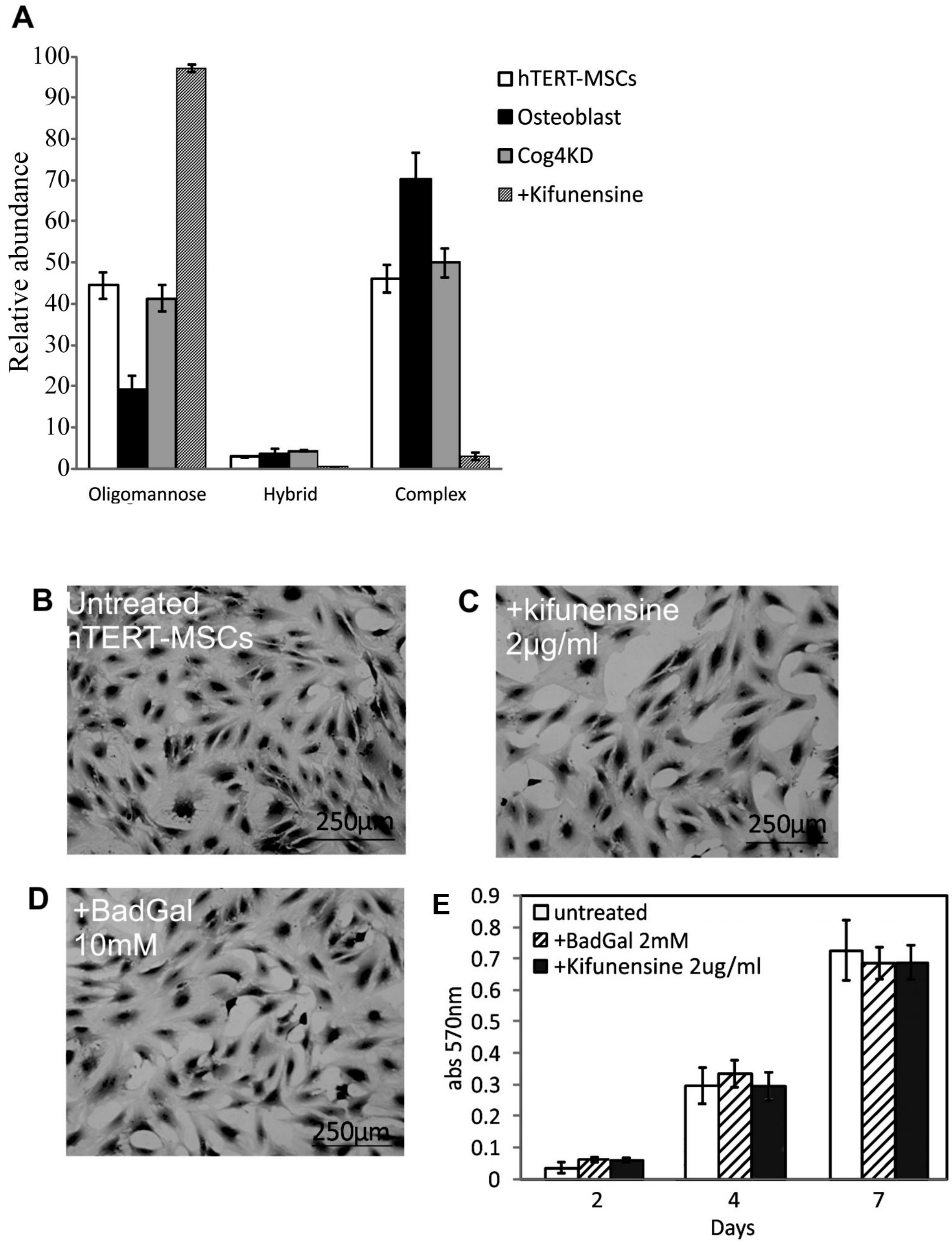

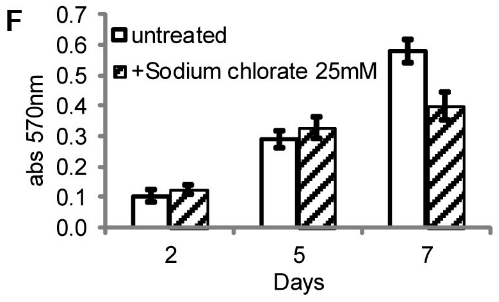

Supplementary Figure 2. Effects of glycan processing inhibitors on glycans and cell proliferation in Y101 hTERT MSCs.
(A) Quantification of glycan classes in the *N*-glycan profiles of WT, Cog4KD and osteogenically differentiated Y101 hTERT-MSCs as well as WT cells treated with kifunensine. Error bars are SEM for n=5 (hTERT-MSC) or n=3 (all others). The WT and osteoblast *N*-glycan quantifications have been published in (Wilson et al., 2016), and are reproduced here for comparison. (B-D) Bright-field images of cells stained with crystal violet following continuous treatment with the indicated inhibitor. (E-F) Results of MTT assays comparing untreated and continuously inhibitor treated cells.

Journal of Cell Science • Supplementary information

Wilson et al.
Supplementary Figure 3.

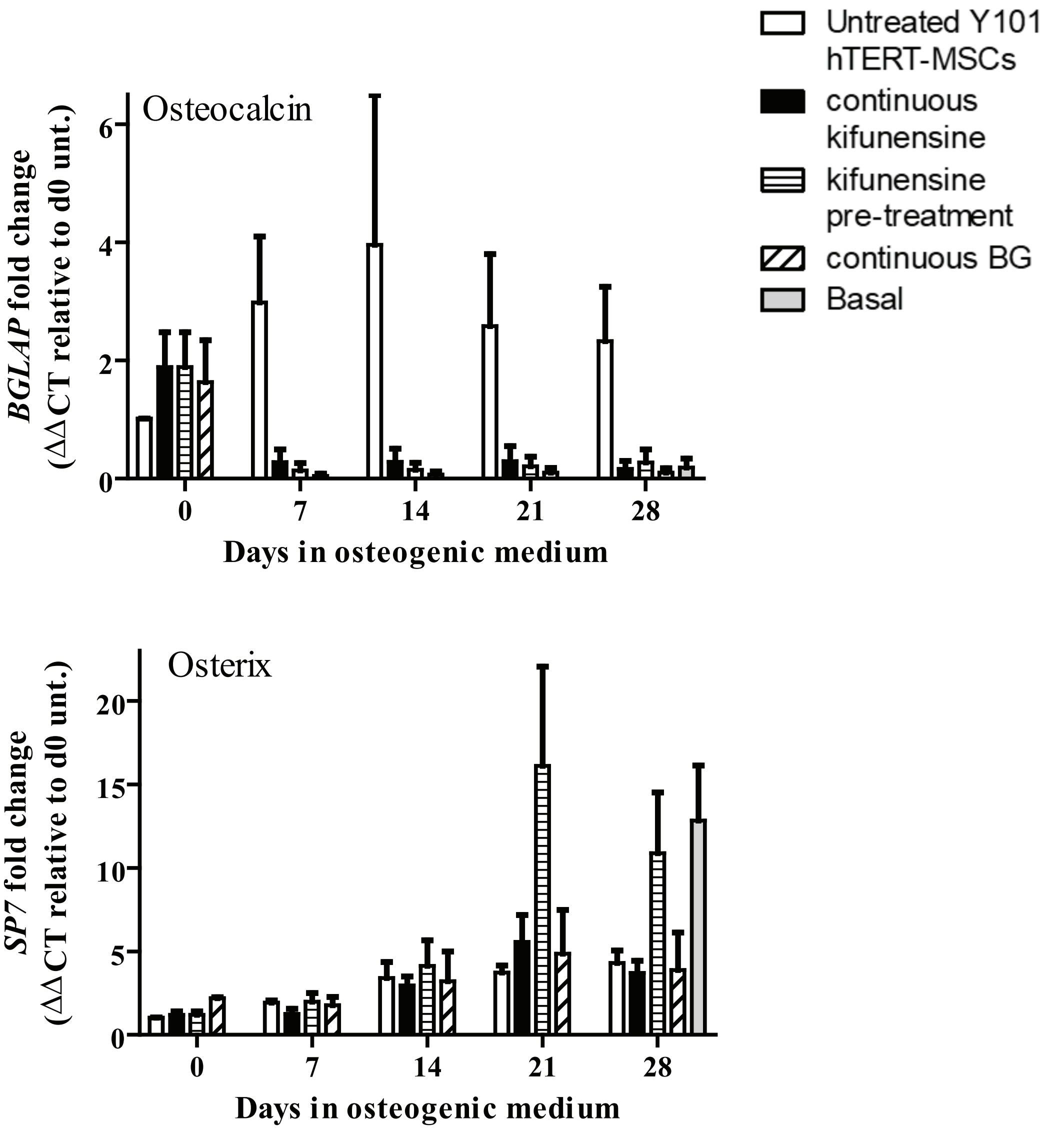

Supplementary Figure 3. Analysis of early and late differentiation markers in drug treated cells during osteogenic differentiation.
Quantitative real-time PCR analysis of mRNA expression levels of the early marker osterix and the late marker osteocalcin in Y101 hTERT-MSCs. Medium was supplemented with the indicated inhibitors two days prior to the start of differentiation, and where indicated continuously kept in the medium until the end of the differentiation experiments. Averages of triplicate measurements were normalized to the day zero control. The error bars are the SEM for three independent measurements, each normalized independently to its own control. None of the differences are statistically significant when the whole datasets are analysed. When samples are compared within a day the untreated samples are statistically significantly different from the drug treated ones on days 7 and 28 for osteocalcin.

Wilson et al
Supplementary Figure 4.

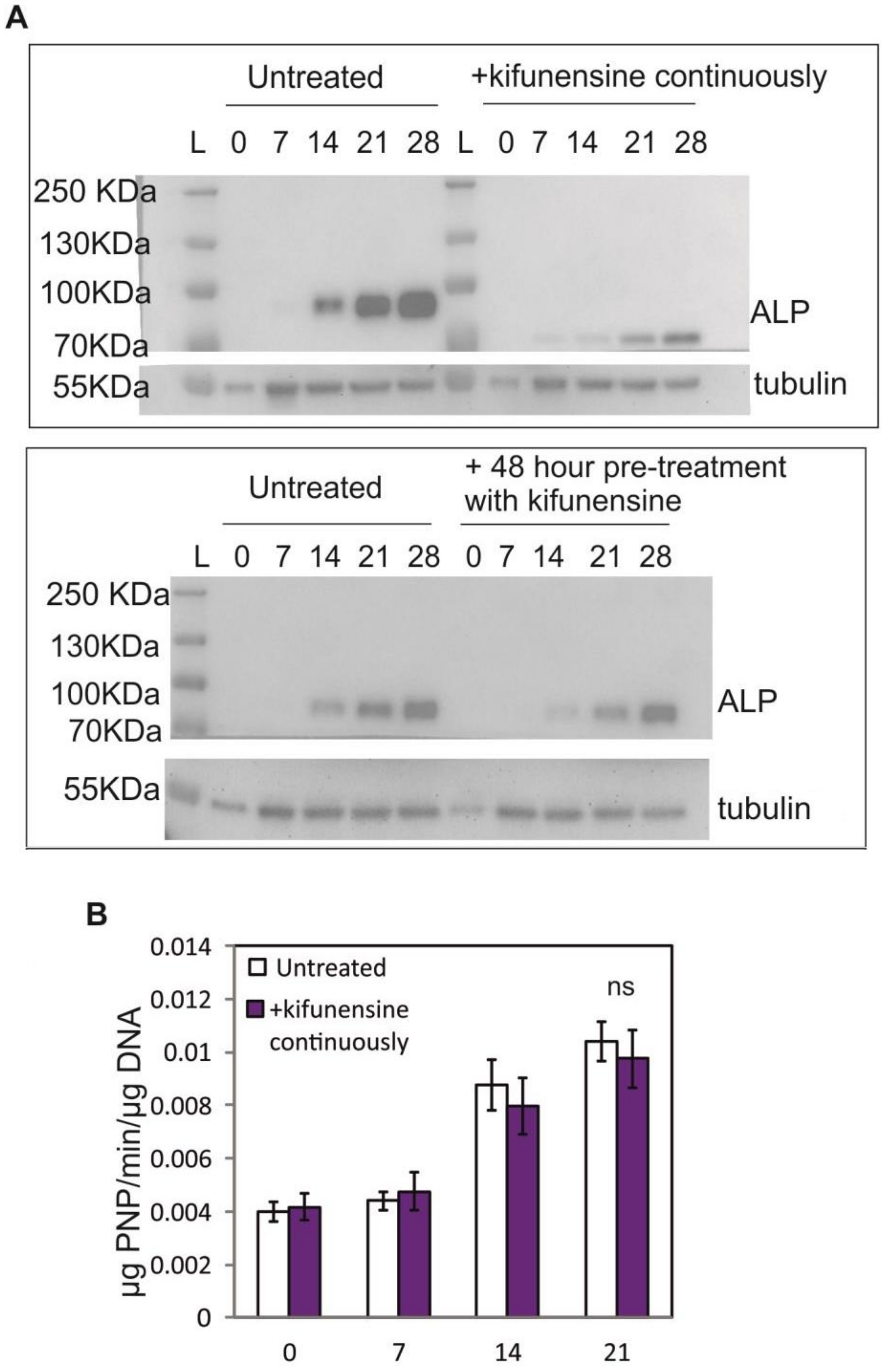

Supplementary Figure 4. Effect of kifunensine treatment on ALP activity and protein levels.
(A) Western blots of cells cultured in osteogenic medium for the indicated time and probed with anti-ALP antibody. Kifunensine treatment was continuous (top) or for 48 h pre-treatment only (bottom). Beta-tubulin was used as a loading control, L = protein molecular weight marker. (B) ALP activity inY101 cells cultured in osteogenic medium ±kifunensine for the indicated time was measured using pNP assay and normalized to DNA content determined using a pico-green assay. One way ANOVA, n=6, ns= non-significant, error bars show standard deviation.

Wilson et al.
Supplementary Figure 5

A

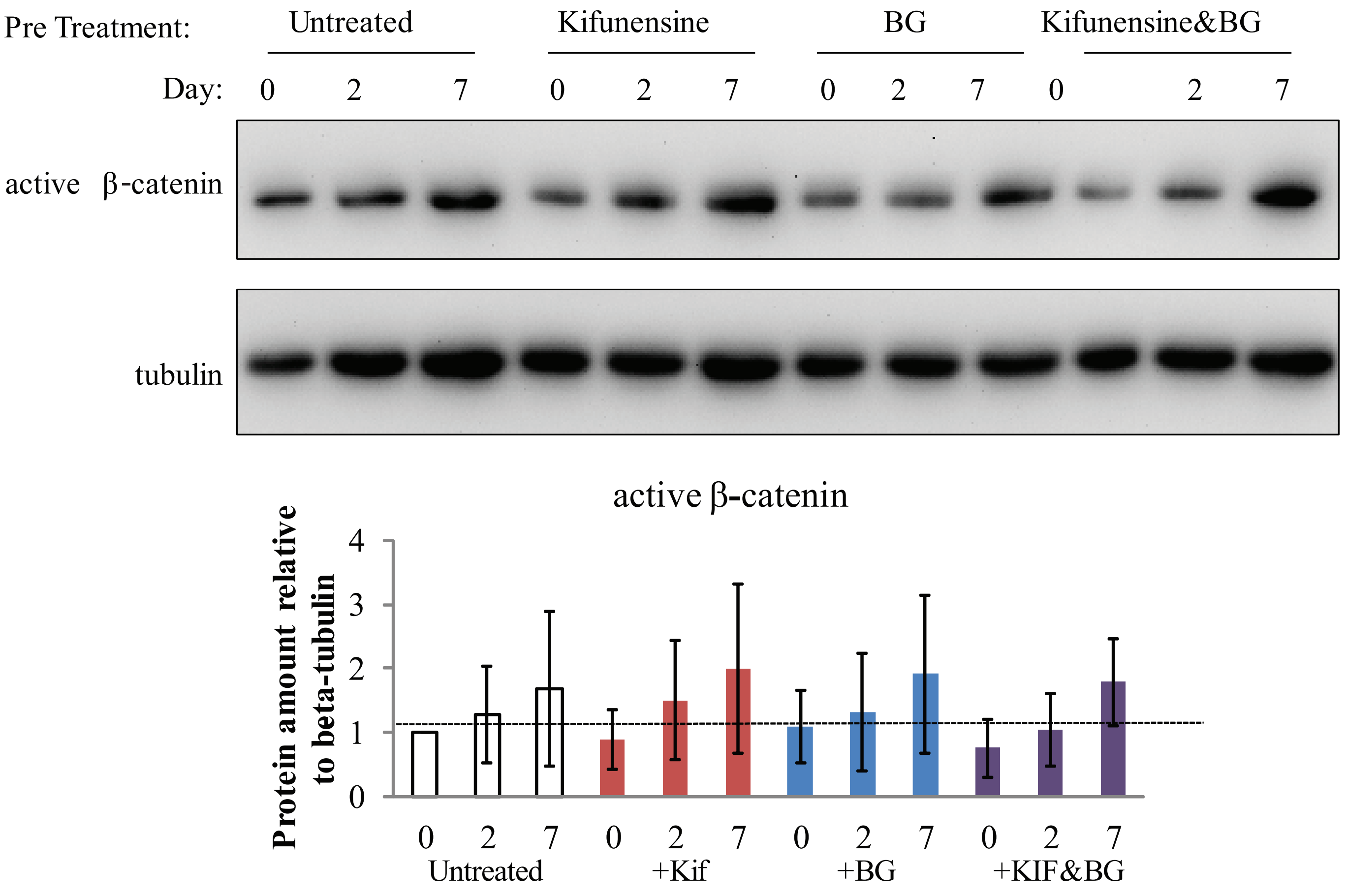

B

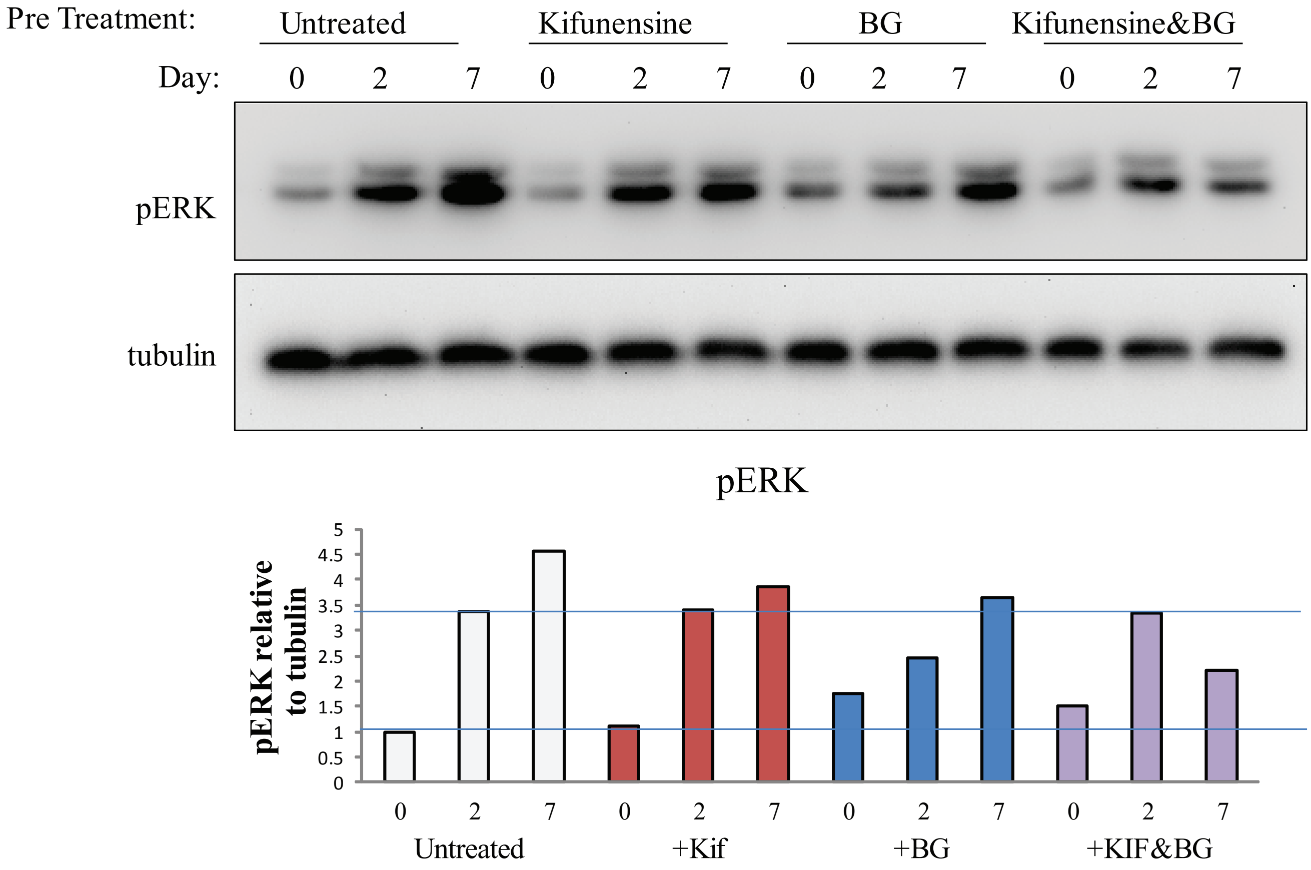

Journal of Cell Science • Supplementary information

Supplementary Figure 5. Wnt/β-catenin and MAPK signalling are unperturbed by the tested glycosylation inhibitions during early osteogenesis.
Cells were grown and blotted as in Fig 5, β-tubulin was used as a loading control. Blots were probed for (A) active β-catenin (n=2) and (B) phosphorylated ERK (n=1).

Supplementary Figure 6. Wortmannin inhibition of PI3K during early osteogenesis.
Y101 hTERT-MSCs were cultured for 6 h in osteogenic medium in the presence or absence of 100 nM wortmannin and the phosphorylation state of Akt assessed (top). pAkt signals were quantified relative to the 0 h time-point run on the same gel following normalization to β-tubulin and total Akt levels. Error bars represent SEM for n=3 (S473) and n=6 (T308).

Wilson et al.
Supplementary Figure 6.

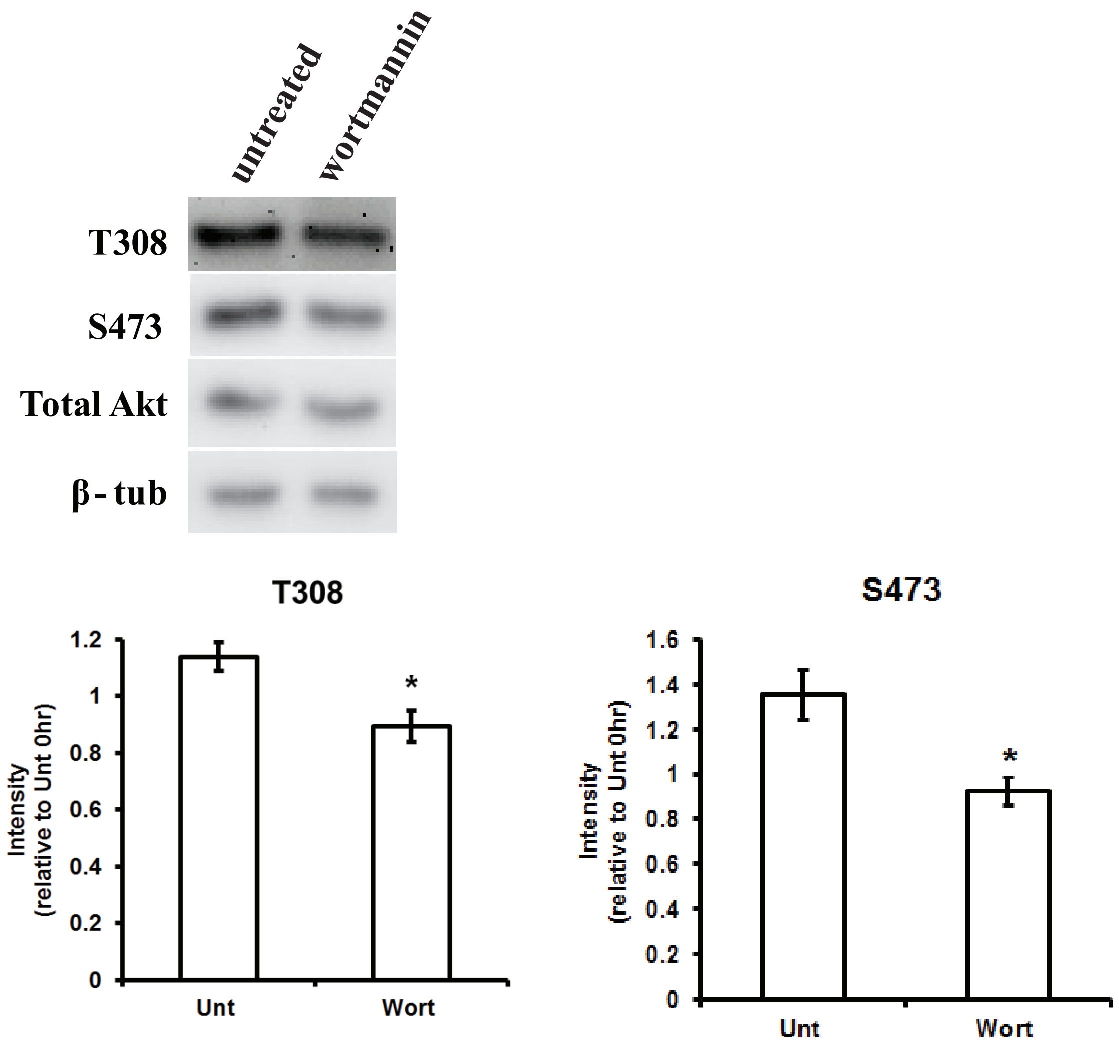

Journal of Cell Science • Supplementary information

Supplementary Table 1. Comparison of averaged glycan abundances for the hTERT Y101 MSC and Cog4KD shRNA1 cells

Click here to Download Table S1
